# Feasibility of a snowball sampling survey to study active surveillance for thyroid microcarcinoma treatment among endocrinologists and surgeons of Brazil

**DOI:** 10.1016/j.bjorl.2022.01.005

**Published:** 2022-02-04

**Authors:** Isabela Matos da Silva, Taísa Quécia da Silva Nogueira, Deborah Nogueira Couto, Paula Cristina Tanajura Meira Lima, Natália Sampaio Carvalho Bonfim, Izadora Gomes Veiga de Sousa, Ana Clara Tosta Telles, Fábio Hecht, Nina Ramalho Alkmim, Gustavo Cancela e Penna, Carolina Ferraz, Eduardo Tomimori, Helton Estrela Ramos

**Affiliations:** a Centro Estadual de Referência em Atenção ao Diabetes e Endocrinologia, Salvador, BA, Brazil; b Universidade Federal da Bahia, Faculdade de Ciências Médicas, Serviço de Endocrinologia, Salvador, BA, Brazil; c Hospital Geral Roberto Santos, Serviço de Endocrinologia, Salvador, BA, Brazil; d Universidade Federal da Bahia, Instituto de Ciências da Saúde, Programa de Pós-Graduação em Processos Interativos de Órgãos Sistemas, Salvador, BA, Brazil; e Universidade Federal do Rio de Janeiro, Instituto de Biofísica Carlos Chagas Filho, Rio de Janeiro, RJ, Brazil; f Faculdade de Medicina da Universidade Federal de Minas Gerais, Belo Horizonte, MG, Brazil; g Irmandade da Santa Casa de Misericórdia de São Paulo, Faculdade de Ciências Médicas da Santa Casa, Unidade de Doenças da Tireóide ‒ Divisão de Endocrinologia, Departamento de Medicina, São Paulo, SP, Brazil; h Instituto da Tireoide, Indatir, SP, Brazil; i Universidade Federal da Bahia, Instituto de Ciências da Saúde, Departamento de Biorregulação, Salvador, BA, Brazil; j Universidade Federal da Bahia, Faculdade de Ciências Médicas, Programa de Pós-Graduação em Medicina e Saúde, Salvador, BA, Brazil

**Keywords:** AS, Active Surveillance, PTMCs, Papillary Thyroid Microcarcinoma, TT, Total Thyroidectomy, HT, Hemithyroidectomy, ND, Nodal Dissection, US, Ultrasound, LT4, Levothyroxine, TH, Thyroid Hormones, IMA, Instant Message Application, Microcarcinoma, Slow-risk thyroid cancer, Active surveillance, Survey

## Abstract

**Objectives:**

This study aims to investigate if a sampling method using virtual networks is feasible to survey AS adoption among this “hard-to-reach” population of Brazilian doctors.

**Methods:**

An online piloted 11-point structured survey questionnaire (designed using Googleforms®) probed the actual treatment patterns for adult patients with PTMCs, including treatment decision‐making nonoperative options, was undertaken between 10 November and 30 November 2020. Participants were reached by the mobile phone Application (APP) and a snowball sampling strategy was used to recruit a total of 4783 members (maximum number of potential reach), which is the total of doctors of the all 21 social media WhatsApp® groups.

**Results:**

From a total of 4783 members (maximum number of potential reach), there were 657 (13.7%) doctors (actual reach) who clicked the web link of the questionnaire, out of whom 512 (10.7%) fully completed the online survey. Among the survey respondents, 361 were endocrinologists (70.5%) and 151 were surgeons (29.5%). Overall, for low-risk PTMCs in an elderly patient, 118 responders (23%) recommend AS, while 390 (76%) recommend immediate surgery as the management, including lobectomy (18.5%) and Total Thyroidectomy (58.2%). The present responders tended to recommend surgery for PTMCs that were located adjacent to the dorsal surface of the thyroid, were multiple, or raised the size during the follow-up.

**Conclusion:**

Using snowball sampling strategy as an innovative route to conduct surveys was feasible and applicable but the rate of response was still very low. Our data also suggests the need to investigate if AS is embraced by Brazilian doctors.

## Highlights

• Active surveillance for low risk papillary microcarcinoma is a strategy to prevent excessive early treatment, among other advantages.
• Snowball sampling strategy is feasible and able in reaching hard-to-reach groups, such as doctors of different specialties, in different work environments or in large countries.
• In Brazil, total thyroidectomy seems to be the most indicated treatment for thyroid papillary microcarcinoma.

## Introduction

Active Surveillance (AS) of indolent malignancies of the thyroid gland is defined as an approach that comprises a period of watching and routine ultrasound examinations, rather than prompt definitive treatments, such as surgery, which are not without risks of meaningful damage, until their disease has advanced to a stage that ensures such interventions.^1^ It might be of service in diminishing exposure of patients to unneeded interventions and costs and, most importantly, could lift the standards of personalized patient care. In preceding years, AS of small-scale papillary thyroid carcinomas, labeled Papillary Thyroid Microcarcinomas (PTMCs), most of which are 10 mm or lesser, has been the focus of discussion worldwide.^2^, ^3^ As a matter of fact, most of PTMCs would never become clinically significant or end in death. If undiscovered, PTMCs will possibly remain asymptomatic and have scant to no impact on a patient’s health, quality of life, or life expectancy.^4^, ^5^

The outcomes are quite attractive and favorable experiences have also been lately reported, chiefly in Japan, Korea, US, Italy, Argentina and Brazil.^6^, ^7^, ^8^, ^9^ However, PTCM has been treated over the past few decades by surgery which can comprise hemithyroidectomy or removing the full gland (total thyroidectomy). The sequela of a total thyroidectomy for all patients is hypothyroidism, requiring lifelong oral replacement of thyroid hormones. The risk of complications raises with the length of surgery and have central lymph-node dissection increases the inherent risk of comorbidities like recurrent laryngeal nerve, leading to permanent hoarseness and damage to the parathyroid glands, which can be either temporary or permanent. The American Thyroid Association (ATA) guidelines supported AS for these patients and favored narrow surgical treatment to hemithyroidectomy.^10^ Notwithstanding, AS studies would demand outcome data from plentiful decades in order to totally calculate the likely percentage of disease progression and disease specific mortality.^11^ A study performed in Brazil showed that two thirds of patients with PTMCs designated the choice for AS to the doctor or wished to know his/her predilection before deciding for AS and that it can be strong endorsed if doctors were persuaded that it is the first option for patients with PTMCs.^8^

In Brazil, an ubiquitous Instant Messenger Applications (IMAs) such as WhatsApp® has been more and more used for clinical case discussion and splitting of relevant journal articles among endocrinologists and head-and-neck surgeons communities portrayed by groups of physicians who partage a tenacious common interest in thyroid diseases, building affinity and continually interacting online.^12^, ^13^, ^14^ Considering this context, WhatsApp® endocrinologists and surgeons communities would be very useful to figure out the beliefs, thinking mechanism and mindset of doctors about treatment of PTMCs.^15^

This was a pilot study that is considered “exploratory research” to investigate if a snowball sampling strategy to conduct survey among Brazilian endocrinologists and surgeons is feasible to investigate doctors’ concerns towards the AS indication for small, low-risk DTC patients.

## Methods

### Study population Recruitment and data collection

An online piloted 11-point structured survey questionnaire (designed using Googleforms®) and concentrated on clinical cases was undertaken between 10 November and 30 November 2020. A snowball sampling strategy, therefore based on a convenience sampling method, was used to recruit doctors. Participants were reached by the mobile phone Application (APP) once a link to the survey was sent to all members of twenty-one WhatsApp® endocrinologists and/or head-and-neck surgeon groups through the WhatsApp® application itself. The questionnaire was about 11 phone screens long, which took ∼5 min to complete.

The invitations to participate in the survey were sent first to doctors’ WhatsApp® groups by researchers, then they were cascaded by purposively inviting key respondents to send the link to their members. A total of 4783 members (maximum number of potential reach), which is the total of doctors of the all 21 social media WhatsApp® groups, could directly receive the invitation.

Questionnaire included five questions and six case‐based clinical vignettes. To be eligible, the physician had to care, at least monthly, for patients with thyroid cancer. This criterion was imposed to ensure some familiarity with diagnostic and treatment decisions. We did not bring up the term active surveillance and evoked operative and non-operative choice for each specific case. Once these parameters were stablished, doctors received a total of four WhatsApp messages on their smartphones with questions corresponding to the questionnaire during the specified time interval (one month). Once analysis revealed that both physician specialties had significant variability with respect to their attitudes, we present the results as a group and identify the specialty. The Review Board approved this prospective study (CAAE: 35513820.7.0000.5662).

The survey comprised sections that are as follows: demographic data including range of age, type of work (categorized as “academic only”, “clinical only”, “academic and clinical”, or “other”), specialty (options included endocrinology, head-and-neck surgery and others), state federation of residence, and how frequent the participant see thyroid cancer patients (options included daily, weekly, monthly and rarely); and clinical case based section with six patients. For this latter section, questions were asked about the best option for each specific case situation (categorized as “Hemithyroidectomy”, “Total Thyroidectomy”, “Total Thyroidectomy and prophylactic central neck dissection” and “Follow up with serial US at 6-months and explain the indolent character of the pathology to patient”).

The first draft of this survey was reviewed by 3 physicians similar to the target group to ensure clarity of the questions and rating scale. No major changes were suggested. A cover letter was attached to the survey explaining its purpose, the investigator’s information, the anonymity of participants, and the confidentiality of the information. No personal identification was requested or stored. The survey was distributed via URL link through Google forms. Participants were informed at the start of the survey about the length of time needed to complete the survey, the investigators and the purpose of the study. Participants were free to continue the survey or to quit at any time. Anonymous data were collected. No incentive was used to reward participants. No patient or public was involved in the design or planning of this study.

### Statistical analysis

Data were imported from the survey website into Microsoft Excel 2016, then converted into the SPSS database. Analyses were conducted using SPSS version 20.0 (SPSS Inc, Chicago, IL, USA). As well as basic descriptive data for all outcomes, results were compared between participants according to their demographic and specialty data. Summary statistics are reported as frequency and percentages. Descriptive statistics, Mann-Whitney test, χ^2^ test and multivariate regression were applied in description and analysis of the variables, where appropriate. Doctors with different specialty, levels of seniority, practicing organizations and geographic regions were considered as subgroups and compared. Logistic regression was used to screen factors relevant to knowledge of clinical use of AS.

## Results

### Physician demographic characteristics

**Figure 1:**
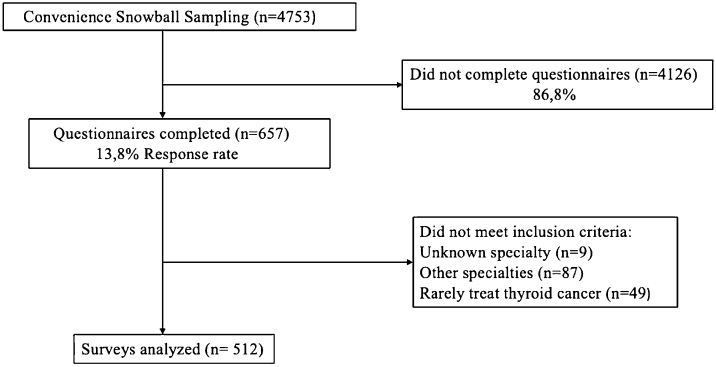
Summary of survey response.

**Figure 2:**
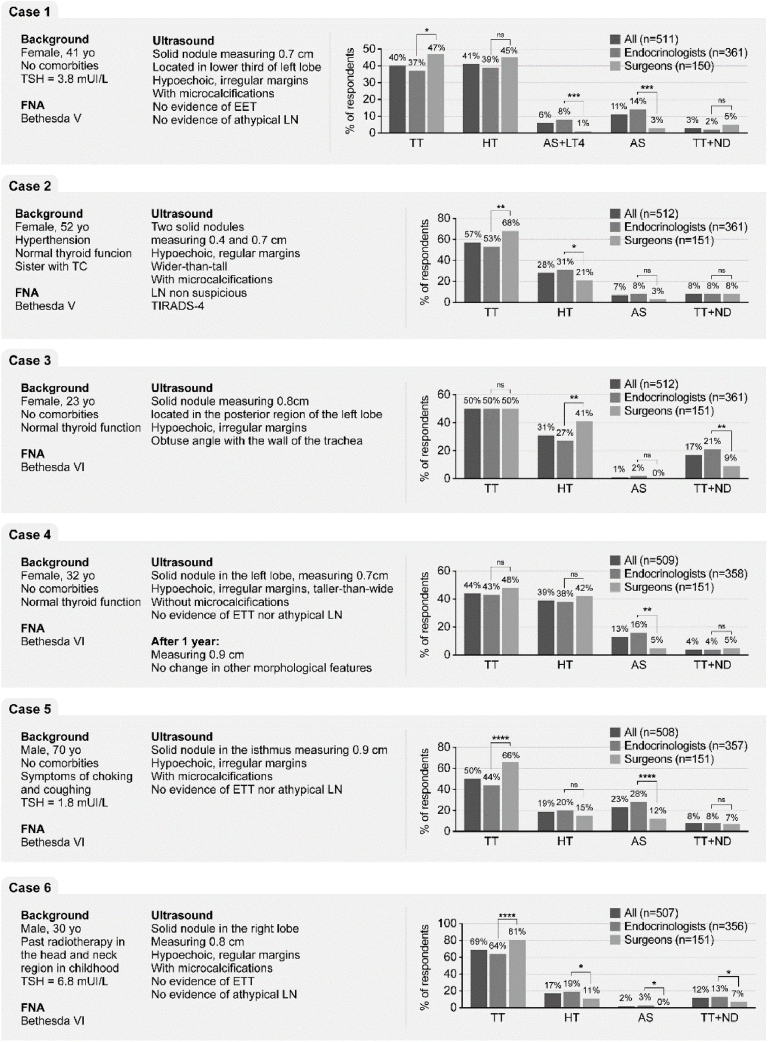
Attitudes of doctors towards management of a patient with nodule suspect or confirmed as small, low-risk papillary thyroid cancer. Six clinical cases involving different scenarios of PMTC in relation to the age of the patients, multinodularity and location of the nodule were proposed to the participants. yo, years old; PTC, Papillary Thyroid Cancer; EET, Extrathyroidal Extension; LN, Lymph Node; TC, Thyroid Cancer; TIRADS, Thyroid Imaging Report and data System; TSH, Thyroid-Stimulating Hormone; TT, Total Thyroidectomy; HT, Hemithyroidectomy; AS, Active Surveillance; TT + ND, Total Thyroidectomy and prophylactic central Neck Dissection.

From a total of 4783 members (maximum number of potential reach), there were 657 (13.7%) doctors (actual reach) who clicked the web link of the questionnaire, out of whom 512 (10.7%) fully completed the online survey (Figure 1, Figure 2). Among the survey respondents, 361 were endocrinologists (70.5%) and 151 were surgeons (29.5%). Demographics of all participants are shown in Table 1.

**Table 1:** Demographic characteristics and background of responders.

	n (%)
**Age (y)**	
< 40	172 (33.6)
41‒60	291 (56.9)
> 60	48 (9.3)
**Experience with thyroid cancer patient**	
Daily	131 (26)
Weekly	213 (42)
Monthly	167 (33)
**Country location**	
Northeast	170 (34)
Southeast	170 (34)
Central Western	41 (8)
North	15 (3)
South	107 (21)
**Practice**	
Private Hospital	336 (66)
Public Hospital	49 (10)
Academic Center	31 (6)

### Treatment decision‐making of suspect thyroid nodules in specifics clinical scenario

In Case 1, in which there was no issue such as age, multinodularity or unfavorable location, only 17% of responders would send the patient to AS. However, AS with or without LT4 prescription were recommended by 22% vs. 4% by endocrinologists and surgeons, respectively (*p* < 0.001) (Fig. 2). Peculiarly, HT appeared as a practically equally indicated procedure if compared with TT (40% vs. 41% respectively) and more surgeons significantly favored TT as the first choice for this patient (47% vs. 37%; *p* < 0.01).

In Case 2, which patient harbored multifocal disease with two microcentimetric ipsilateral thyroid nodules suspect of malignancy, TT was largely chosen by 57% of doctors, followed by HT (28%). Indeed, 8% of doctors would also advocate TT + ND vs. only 7% who proposed AS. One more time, surgeons significantly chosen TT when compared with endocrinologists (68% vs. 53%, *p* < 0.001).

The Impact of malignant nodule location on physician’s decision was investigated with the clinical Case 3 where the nodule was posteriorly located in a young patient. In this scenario TT was equally preferred by 50% of endocrinologists and surgeons, whereas significantly more surgeons preferred HT (41% vs. 27%, *p* <  0.01) and a small minority of doctors chosen AS.

The influence of patient age in decision-making was additionally debated thorough the Case 4, where a young female patient presented a confirmed PTMC already in AS for one year but presented a 2 mm increased in nodule diameter. 44% and 39% of physicians preferred to stop the AS and perform TT and HT, respectively. No differences between surgeons and endocrinologists were observed when surgery was the option. However, significantly more endocrinologists preferred to continue the AS (16% vs 5%, *p* <  0,001).

Age greater than 60-years old seems to be the best accepted situation for AS, once 23% of all doctors, in clinical Case 5, preferred AS. Still, more endocrinologists preferred AS than surgeons (28% vs. 12%; *p* <  0.0001). Surgeons significantly preferred to perform TT in a 70-years-old man with a PTMC (66% vs. 44%, *p* <  0.0001).

In the last case, a young patient previously exposed to radiation therapy developed a TC and high TSH. Again, TT was mostly chosen by surgeons (81% vs. 64%, *p* <  0.001). In contrary, AS was chosen by 19% of endocrinologists vs. 11% of surgeons (*p* <  0.01).

In summary, for five of the six cases, TT was always the first choice for, independent if the respondent was surgeon or endocrinologist. Also, fewer than 25% of respondents recommended AS as the first choice, even for an undoubtedly case, without any additional risks.

## Discussion

To our knowledge, this is the largest survey study focused on physician’s clinical conduct regarding PTMC management in Brazil. The use of social media as one of the recruitment strategies associated with snowball sampling strategy might be particularly effective in reaching hard-to reach groups, such as doctors of different specialties, in different work environments and cities, especially in a large country as Brazil. One third of all responders were less than 40-years old. No difference in respect of age was demonstrated between adopters and non-adopters of AS, except in Cases 2, 3 and 6. Our strategy could thus increase the probability of attracting younger participants, who will be the future generation to continue the follow-up of the patients under AS. Our data shows differences between endocrinologists and surgeons with regard to their choices on management of PTCMs patients in a variety of clinical scenarios. Overall, surgeons were significantly less likely than endocrinologist to adopt AS in the great majority of cases.

Based on answers related to Case 1 management, it is possible that most doctors still perform Fine-Needle Aspiration Cytology (FNAC) for suspicious nodules ≤10 mm and do not propose AS as the management choice of PTMC. However, recently, a Brazilian experts opinion panel was published and AS in adult patients, with low risk PTMC, was recommend as the first-line management.^16^ Nonetheless, a tendency towards acceptance AS was shown in elderly patients, while nodule posteriorly located, multicentricity and previous exposition to radiation negatively influenced the adoption of AS, in our survey. In contrast to our results, a recent survey study from Unites States noticed that AS was preferred by more than half of the responders.^17^, ^18^ Similarly, the management of PTMCs in Japan was recently surveyed and more than 50% of low-risk PTMCs were on AS.^5^ If surgery is an option, generally HT has been considered as the best option for patients with PTMC who are not candidates for AS because of age, proximity of the tumor to the trachea or recurrent laryngeal nerve, or because they opted for surgery.

A recent study, which surveyed North American endocrinologist, surgeons and otolaryngologists, found that years of practice did not have significant effect on considering AS an appropriate option for some patients with thyroid cancer. Curiously, in this study, the majority (76%) of responders believed that AS was a possible management, but only 44% used it in their practice. In contrast, among physicians who believed that AS was possible in some cases but did not use in their practice, those with more years of practice were less likely to use AS.^19^ In the light of these results, strategies to improve awareness about best practices for PTMC should be highly inclusive, covering young future specialists and those with years of clinical practice.

This study must be considered in the context of its limitations: (i) Only ∼11% of potential target population have completed the questionnaire, (ii) This survey was conducted through the WhatsApp and the data integrity cannot be completely confirmed, (iii) It is a self-report survey, consequently it is not possible to confirm if the data collected through the survey reflects the clinical practice, (iv) Included physicians mainly from two regions of Brazil, who belong to the endocrinologist and head-and-neck surgeons ‘communities. Indeed, every survey is subject to social desirability bias, which reflects a tendency to answer self-report questions correctly, to be viewed favorably by others, even considering that the data was collected in an anonymous way. Therefore, it certainly does not reach all Brazilian physicians that treat PTMC, possibly causing a strong selection bias. We are aware that the response rate in the present survey does not covers the majority of endocrinologists and surgeons that treat thyroid cancer in Brazil and, consequently, the answers collected in this study does not reflect the real clinical practice of the responders, in concern of the management of PTMCs.

## Conclusion

Using snowball sampling strategy to conduct a survey about management of PTMCs and AS adoption among Brazilian doctors was feasible and applicable but the rate of responders was still very low. Our results pinpoint that AS should be further investigated as a non-invasive established practice in Brazil and a nationwide, long-term continuous case accumulation survey system should be performed.

## Conflicts of interest

The authors declare no conflicts of interest.
